# Visual Enhancement of Illusory Phenomenal Accents in Non-Isochronous Auditory Rhythms

**DOI:** 10.1371/journal.pone.0166880

**Published:** 2016-11-23

**Authors:** Yi-Huang Su

**Affiliations:** Department of Movement Science, Faculty of Sport and Health Sciences, Technical University of Munich, Munich, Germany; Universiteit van Amsterdam, NETHERLANDS

## Abstract

Musical rhythms encompass temporal patterns that often yield regular metrical accents (e.g., a beat). There have been mixed results regarding perception as a function of metrical saliency, namely, whether sensitivity to a deviant was greater in metrically stronger or weaker positions. Besides, effects of metrical position have not been examined in non-isochronous rhythms, or with respect to multisensory influences. This study was concerned with two main issues: (1) In non-isochronous auditory rhythms with clear metrical accents, how would sensitivity to a deviant be modulated by metrical positions? (2) Would the effects be enhanced by multisensory information? Participants listened to strongly metrical rhythms with or without watching a point-light figure dance to the rhythm in the same meter, and detected a slight loudness increment. Both conditions were presented with or without an auditory interference that served to impair auditory metrical perception. Sensitivity to a deviant was found greater in weak beat than in strong beat positions, consistent with the *Predictive Coding* hypothesis and the idea of metrically induced illusory phenomenal accents. The visual rhythm of dance hindered auditory detection, but more so when the latter was itself less impaired. This pattern suggested that the visual and auditory rhythms were perceptually integrated to reinforce metrical accentuation, yielding more illusory phenomenal accents and thus lower sensitivity to deviants, in a manner consistent with the principle of inverse effectiveness. Results were discussed in the predictive framework for multisensory rhythms involving observed movements and possible mediation of the motor system.

## Introduction

Musical rhythms are often more complex than a simple metronome, and yet most of us can move effortlessly to a regular beat in music [1]. The fact that we can move to a beat, instead of moving to every sounding event, relies on the perceptual differentiation between more and less accented metrical periodicities [2] and synchronization with the former [3]. Regular accentuation of events in a rhythm to yield a metrical structure is known as metrical accentuation. Metrical accentuation may–but needs not–originate from physical saliency of the sounds, such as increased loudness [4] or an additional beat superimposed on regular temporal positions [5]. These accents are termed *phenomenal accents* [6,7] to relate to the physical accentuation of the sounds. On the other hand, a listener can also perceive metrical accents solely based on temporal grouping of successive, identical sounds separated by various intervals [8]. If such temporal accents arise regularly, the rhythms are considered to be metrically simple [9,10] or strongly metrical [11].

Events coinciding with metrically accented and less accented positions are often perceived as stronger and weaker beats, respectively [9]. Two different hypotheses have been proposed to explain the perceptual mechanisms of these events, which make opposite predictions [12]. In one hypothesis, which is based on the Dynamic Attending Theory (“DAT”) [13], attention is modeled as oscillatory attentional energy being entrained to hierarchical periodicities in musical rhythm, and enhanced attention is temporally allocated to the accented (or strong beat) positions. As a result, events coinciding with these positions receive more attentional resources and are better processed. Findings supporting this hypothesis generally show faster reaction to, or better detection of, targets occurring in these temporal positions than in others [14–16]. Besides, attention can be entrained in one modality (often audition) to facilitate target detection in the same [7,12,17] or in a different modality (often vision) [14–16], as long as the temporal structures of the two sensory streams correspond to each other.

The other hypothesis adopts the Predictive Coding (PC) approach [18], positing rhythm perception as using the musical meter to form top-down temporal expectations (or “*priors*” in Bayesian terms, [19]), against which the occurrences of unfolding events are compared. Violation of the expectation generates a prediction error, which is then used to correct the expectation. With this mechanism, events are more anticipated to occur in the metrically accented positions. Consequently, events coinciding with more accented positions elicit less prediction error and thus *less* response. In connection with this approach, the metrical accentuation of a rhythm may also induce “illusory phenomenal accents”, a term coined in an earlier study [7]. Namely, events coinciding with strong beat (or accented) positions may be subjectively perceived as louder than those of the same physical loudness in weak beat (or less accented) positions. As discussed in [7], illusory phenomenal accents could arise as a result of top-down modulation of the auditory percepts by the mental construct of musical meter, in line with the PC proposal. In addition, they could also be associated with a bias in perception because strong beats are *expected* to be more salient. One possibility is that listeners (implicitly) assume, or expect, that events in strong beat positions should be louder, which influences how they judge the loudness of these events: A real loudness increment may become less noticeable in strong beat positions, as a louder event fits the expected saliency in these positions and is thus not perceived as (or judged to be) deviating from the baseline. In other words, a louder strong beat is not heard as louder, as both the perception and the decision criterion could be modulated by metrical saliency. Results supporting the PC hypothesis and the illusory phenomenal accents have been demonstrated in deviant detection within the same modality: For example, an increased loudness in an auditory rhythm is more noticeable in metrically weak than in metrically strong positions [12,20], as the former is less expected and yields a greater prediction error, or that the latter induces more illusory phenomenal accent to mask the real loudness increment.

While both DAT and PC hypotheses have received empirical support, they are often tested using different tasks and paradigms, and several issues remain to be solved. First, regarding deviant detection of a loudness increment, the DAT hypothesis predicts better detection in metrically accented positions, whereas the PC hypothesis predicts better detection in metrically unaccented (or less accented) positions. An earlier study investigating detection of a loudness deviant in different metrical positions found only results in favor of DAT [7]. Another study, however, found a larger cortical response to a loudness increment in weak beat positions, in line with PC [20]. A more recent study examined the predictions under these two hypotheses using both reaction time and EEG measurements [12], whose results appear to offer partial support to both. As such, there still seems to be no consensus as to which hypothesis better explains perception as a function of metrical position. Secondly, the aforementioned studies employed only isochronous auditory stimuli, and it is unclear whether and how the results can be generalized to non-isochronous auditory rhythms, which would be closer to real music. Thirdly, while DAT was originally developed in the auditory modality, it has also been tested using a bimodal paradigm, e.g., entraining attention with an auditory metronome while imposing a visual detection task [14–16], thus establishing the cross-modal nature of attentional entrainment. By contrast, studies examining PC mechanisms in the context of musical rhythm have thus far only adopted an auditory task [7,12,20], and it is unclear whether temporal prediction can also be shared between modalities. While recent research demonstrates multisensory (audiovisual, “AV”) rhythm perception with visual movement stimuli [21–23], it has been conducted only with respect to DAT. As such, the possibility of multisensory prediction–as often proposed for speech [24]–remains an intriguing point of investigation in a musical context.

The present study aimed to address these issues using a multisensory task. The following questions were asked: (1) When listening to non-isochronous auditory rhythms with clear metrical accents, would sensitivity to a loudness increment be greater in strong beat (i.e., supporting DAT) or in weak beat positions (i.e., supporting PC)? (2) Previous findings show that a periodic human bouncing movement can induce a visual beat to enhance auditory beat perception [21,22]; besides, visual rhythms of more complex metrical structures can be perceived in realistic point-light dance movements [25]. Given the intrinsic link between the rhythms of music and dance [26], could auditory metrical perception be enhanced by simultaneously observing dance movements with a comparable metrical structure? Would the multisensory effect be reflected in deviant detection, if either DAT or PC hypothesis were supported? (3) Finally, a previous study [22] showed that AV beat perception followed (to a considerable extent) a multisensory integration profile, with greater visual enhancement when the auditory beat perception was more disrupted. This pattern–namely, more multisensory benefit when the unisensory response is weaker–is known as the *Principle of Inverse Effectiveness* [27,28], which in humans is often examined in AV speech perception [29]. The present study asked whether such an integration profile would be observed when auditory rhythms were paired with the point-light dance movement. Depending on whether the DAT or PC hypothesis was supported, different patterns of results were predicted (formulated below).

The present study borrowed the design of a previous one that dealt with multisensory beat perception [22]. In that study, a strongly metrical auditory rhythm was presented either on its own (auditory only, termed “A”), or accompanied by a periodically bouncing humanlike figure that served as a visual beat (termed “AV”). Both conditions were presented with or without an auditory interference. The interference was an isochronous sequence whose period was polyrhythmic to the beat of the attended auditory rhythm, thus disrupting beat perception of the rhythm. There were four increasing levels of auditory interference, implemented as four increasing tempi (and thus increasing polyrhythmic complexity when combined with the attended rhythm). Participants detected a temporal perturbation in the third repetition of the rhythm. It was found that the visual beat improved auditory detection, and more so when the auditory performance was worse, consistent with the inverse effectiveness principle. In that task, however, the temporal deviant always occurred in a strong beat position, and thus the results could only be accommodated in the DAT framework.

**Fig 1:**
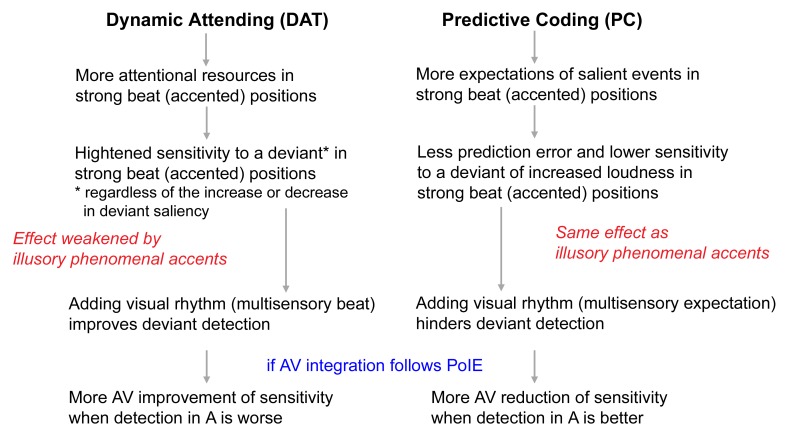
Diagram of the predictions for deviant (loudness increment) detection under each hypothesis. *A*: auditory; *AV*: audiovisual; *PoIE*: principle of inverse effectiveness.

In the present task, participants listened to three repetitions of a strongly metrical rhythm either on its own (A), or accompanied by a point-light figure [30] performing the basic steps of the *Charleston* dance (AV). The rhythm of this dance corresponded to a 4/4 musical meter, matching that of the auditory rhythm, whereby the leg movements marked the beat (see [25] for detailed descriptions of the movement and its visual rhythm perception). The A and AV conditions were presented with or without the same auditory interferences as implemented in [22]. Instead of a temporal perturbation, here participants detected a slight loudness increment in the third repetition. The deviant could coincide with either a strong beat or a weak beat position. Sensitivity (*d’*) to the deviant was taken as the main index of the detection task, and response criterion (*C*) was also used to indicate potential biases in the decision level (see Data analysis). Different hypotheses were formulated regarding these two measures to verify whether results supported the DAT or the PC account (see also **Fig 1** and **Table 1** for an overview):

**Table 1:** Overview of the main anticipated effects on sensitivity (*d’*) and response criterion (*C*) under the Dynamic Attending Theory (DAT) and the Predictive Coding (PC) hypotheses.

Effect	Dynamic Attending (DAT)	DAT + Illusory phenomenal accents	Predictive Coding (PC)	PC + Illusory phenomenal accents
**Metrical position**	*d’*_strong beat_ > *d’*_weak beat_	*d’*_strong beat_ ~ = *d’*_weak beat_	*d’*_weak beat_ > *d’*_strong beat_	*d’*_weak beat_ > *d’*_strong beat_
**Modality**	*d’*_AV_ > *d’*_A_	*d’*_AV_ ~ = *d’*_A_	*d’*_A_ > *d’*_AV_	*d’*_A_ > *d’*_AV_
		*C*_AV_ ~ = *C*_A_		*C*_AV_ > *C*_A_
**AV integration profile**	Greater *d’*_AV–A_ for lower *d’*_A_	No clear integration profile	Greater *d’*_A–AV_ for higher *d’*_A_	

*A*: auditory; *AV*: audiovisual. Note that, regarding *d’*, the presence of illusory phenomenal accents would counteract the effects predicted by DAT while yielding effects consistent with those predicted by PC.

DAT hypotheses: Sensitivity to a loudness increment should be greater in strong beat than in weak beat positions. However, if heightened attention in strong beat positions also led to the event to be perceived as louder, giving rise to illusory phenomenal accents, this effect might weaken or cancel out the effect of metrical position (cf [12]); i.e., sensitivity in strong beat positions would then be comparable to that in weak beat positions or would be only slightly greater than the latter. The presence of the visual rhythm (dance movement) should improve auditory deviant detection [22], which should be more evident in strong beat positions. As DAT does not associate enhanced attentional resources with criterion changes in the decision level, a comparable response criterion was expected with or without the visual rhythm. Finally, if the AV streams were perceptually integrated, then the multisensory response profile would follow the principle of inverse effectiveness: namely, greater visual enhancement (performance in AV relative to A) when the auditory performance was more impaired by the interference.

PC hypotheses: Sensitivity to a loudness increment should be greater in weak beat than in strong beat positions. The illusory phenomenal accents, induced by the metrical structure, would also lead to better detection of a loudness increment in weak beat than in strong beat positions. If the visual rhythm strengthened auditory metrical perception and enhanced illusory phenomenal accents, it should further attenuate sensitivity to a loudness increment. Besides, as illusory phenomenal accents may result from the expectations of increased loudness in accented positions, they could also influence biases in the decision level. As such, more visually-induced illusory phenomenal accents would be associated with a more conservative response criterion (i.e., the increase in loudness needed to exceed a greater threshold in order to be perceived as louder). Finally, the profile of AV integration in this case, according to the principle of inverse effectiveness, would manifest as more *reduction* of sensitivity (i.e., a greater extent of illusory phenomenal accent) in AV, compared to A, when sensitivity was greater (i.e., less illusory phenomenal accent) in A.

## Method

### Participants

Twenty young, healthy volunteers (five male, mean age 24 years, SD = 3.1) took part in this experiment. Participants were naïve of the purpose, gave written informed consent prior to the experiment, and received an honorarium of 8 € per hour for their participation. Participants were not pre-screened for music or dance training, which ranged from zero to thirteen and zero to twenty years for each (all amateurs). Sixteen and eleven participants had trained in music and dance (none in swing dance), respectively, amongst whom nine had trained in both. The mean duration of music and dance training was 6.5 years (SD = 4.2) and 4.9 years (SD = 5.8). The study had been approved by the Ethic Commission of Technical University of Munich (Ethikkommission der Medizinischen Fakultät der Technischen Universität München), and was conducted in accordance with the ethical standards of the 1964 Declaration of Helsinki.

### Stimuli and materials

#### Visual stimuli

The visual stimuli consisted of a human point-light figure performing the basic steps of the *Charleston* dance in one tempo. The stimuli had been generated by recording a swing dancer performing these steps using a 3-D motion capture system (Qualisys Oqus, 8 cameras at a sampling rate of 200 Hz, with 13 markers attached to the joints, [30]), paced by a metronome with an inter-beat interval (IBI) of 400 ms. The stimuli were taken from a larger set of point-light dance movements used in two recent studies [25,31], where the stimuli preparation and construction were reported in detail. The description here will thus be brief.

**Fig 2:**
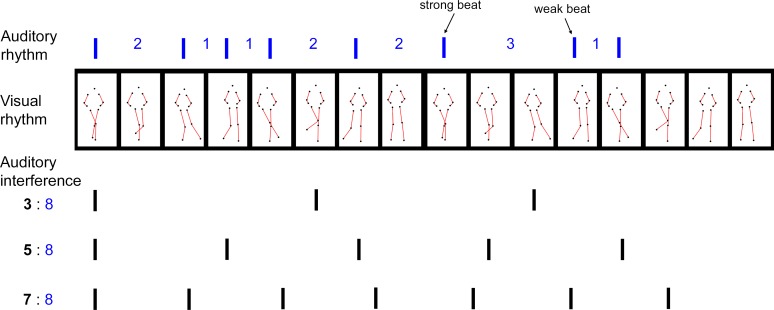
Illustration of the auditory and visual stimuli in the task. *1*^*st*^ *row*: One cycle of the auditory rhythm. Vertical blue bars represent individual sounds, and the numbers indicate the interval ratios between successive sounds, 1 being the smallest interval (400 ms). *2*^*nd*^ *row*: Two cycles of the modified *Charleston* dance as the visual rhythm, matching the length of one auditory rhythm. The dance movement was presented as point-light motion, and the frames represent the postures at each pulse. The auditory strong beats correspond to the leg movements in the odd frames. To ease visualization, the colors of the markers and the background are inverted, and red lines connecting the joints are drawn here, which did not exist in the visual stimuli. *Last three rows*: The three conditions of the auditory interference sequence, whose beat periods (in black bold numbers) are polyrhythmic to the beat of the auditory rhythm (blue numbers). Vertical black bars represent individual sounds.

One cycle of the dance corresponded temporally to eight metronome beats. To present the dance movement continuously, the same cycle was looped (see **S1 Video**). Within a cycle, the trunk bounced vertically at every beat (beat 1 to 8) while the legs moved laterally at every second beat (left leg swinging at beat 1 and 3, and right leg at beat 5 and 7), yielding two levels of periodicity that were related to each other metrically. The figure performed these movements mostly in place without moving the whole body back and forth. As a departure from the authentic *Charleston* dance, where the arms would also swing symmetrically at every second beat, in the present stimuli the arms remained still with the hands placed above the hips throughout the cycle (**Fig 2**, 2^nd^ row). This manipulation was intended to induce a clear visual metrical representation through the trunk and the leg movements only (see [31]).

The 3-D motion data of the dance were presented as a point-light display on a 2-D monitor, using routines of Psychophysics Toolbox version 3 [32] in Matlab ® R2012b (Mathworks). The function *moglDrawDots3D* allowed for depth perception in a 2-D display. The point-light figure was represented by 13 white discs against a black background, each of which subtended 0.4 degrees of visual angle (°). The whole figure subtended approximately 5° (width) and 12° (height) when viewed at 80 cm. The point-light figure was displayed facing the observers, in a configuration as if the observers were watching from 20° to the left of the figure, which served to optimize depth perception of biological motion in a 2-D environment.

#### Auditory stimuli

Two types of auditory stimuli were generated, similar to those used in a previous study [22]: the rhythms, and the interference sequences. The rhythms were ten of the standard patterns employed in [22], which were a subset of the ‘metric simple’ rhythms developed by Grahn and colleagues (see [9]). These rhythms yielded a clear sense of beat in a duple meter by means of temporal accents [8]. The ten rhythms used here all consisted of eight short, discrete sounds in succession that bordered seven empty intervals of different durations (**Table 2**). The sounds had a synthesized timbre of the instrument ‘clave’, each lasting 43 ms (generated by Logic 8 Express, Apple Inc. California). The intervals were related by integer ratios of 1:2:3:4 in various combinations and orders, with the smallest interval being 400 ms (**Fig 2**, 1^st^ row). A silent interval of 800 ms was added to the end of every rhythm, so that an entire pattern corresponded to two measures of a 4/4 musical meter, equaling 16 times the smallest interval (i.e., the length of 16 pulses). The interference sequence consisted of isochronous tones of a synthesized ‘bongo’ sound, each lasting 50 ms. The sequences were generated in three different tempi, whose periods formed the following polyrhythmic ratios to the underlying beat of the rhythms: 3:8, 5:8, and 7:8 (8 being that of the rhythms). The IBI of each interference sequence was thus 2133.33 ms, 1280 ms, and 914.29 ms, respectively (**Fig 2**, last three rows). The interference sequences were slightly attenuated (5 dB) relative to the rhythms. See supplementary material for examples of an auditory rhythm (**S1 Audio**) and the rhythm paired with the three interference sequences (**S2, S3 and S4 Audios**).

**Table 2:** Summary of the ten auditory rhythms presented in the experiment.

Rhythm Pattern	Event/Silence Composition	Deviant Position	Deviant preceded by silence
**1123122**	1110**1**00110101000	5 or 8	Both
**2113113**	1011**1**00111001000	5 or 8	Weak beat
**1122114**	1110**1**01110001000	5 or 8	Strong beat
**1123113**	1110**1**00111001000	5 or 8	Both
**3121113**	1001**1**01111001000	5 or 8	Neither*
**2112231**	10111010**1**0011000	9 or 12	Both
**3141111**	10011000**1**1111000	9 or 12	Strong beat
**4111131**	10001111**1**0011000	9 or 12	Weak beat
**4221111**	10001010**1**1111000	9 or 12	Strong beat
**1111431**	11111000**1**0011000	9 or 12	Both

*1*^*st*^ *column*: the interval ratios of each rhythm pattern. *2*^*nd*^ *column*: each rhythm as depicted by the 16 pulse positions, whereby 1 and 0 represent positions occupied by an event and by silence, respectively. *3*^*rd*^ *column*: pulse positions of a possible deviant in a strong beat (5 and 9) or weak beat (8 and 12) position. For each rhythm there was only one combination of the strong beat and weak beat deviant positions as indicated in this column. The deviant positions are also underlined in the 2^nd^ column, with strong beat positions marked additionally in bold. *4*^*th*^ *column*: the acoustic context preceding both deviant positions in each rhythm. This column shows which deviant position in the respective rhythm was preceded by a silent interval (i.e., at least one “zero” preceding the underlined event in the 2^nd^ column).

* In this rhythm both deviant positions were preceded by an event.

### Procedure and design

The stimuli and the experimental program were controlled by a customized Matlab script and Psychtoolbox version 3 routines running on a Mac OSX environment. The visual stimuli were displayed on a 17-inch CRT monitor (Fujitsu X178 P117A) with a frame frequency of 100 Hz at a spatial resolution of 1024 × 768 pixels. Participants sat with a viewing distance of 80 cm. Sounds were delivered by closed studio headphones (AKG K271 MKII).

The task was similar to that in [22]. Participants self-initiated each trial by pressing the space key. A trial started with a fixation cross in the center of the screen for 1000 ms, followed by three repetitions of an auditory rhythm, during which the number ‘1’, ‘2’, and ‘3’ appeared on the screen accordingly as a reminder. The rhythm was presented either on its own, or accompanied by an interference sequence at one of the three aforementioned tempi. Because of the polyrhythmic relation between the rhythm and the interference sequence, the interference tones never coincided with any, except the first, of the rhythm tones. Each of the four auditory conditions (i.e., one without interference and three with) was presented in two visual conditions: without visual stimuli, or with the point-light figure dancing the *Charleston* steps throughout the three rhythm repetitions. If present, the dance movement always had the same tempo as the auditory rhythm and was synchronized with it. In two thirds of all the trials, there was a loudness deviant in the third repetition of the auditory rhythm, such that one of the rhythm tones was 3dB louder than the others [7,12]; in the other one third of the trials there was no deviant. The deviant could occur in a metrically strong beat or a metrically weak beat position with equal frequency. The former coincided with either the 5^th^ or the 9^th^ pulse position (equally often), whereas the latter coincided with either the 8^th^ or the 12^th^ pulse position (equally often) [14]. See **Table 2** for a complete list of deviant positions in all the rhythms. Participants were instructed to attend to the auditory rhythm and the dance movement (if present), and to detect in every trial whether one of the rhythm tones in the third repetition was louder than the others (**Fig 2**). Participants gave their response by pressing one of the two pre-defined keys (deviant present / deviant absent). No feedback was given.

The experiment consisted of the following conditions: 2 (rhythm modality: A, AV) × 4 (auditory interference: no interference, 3:8, 5:8, 7:8) × 3 (auditory deviant: no deviant, deviant on strong beat, deviant on weak beat). The total trials were presented in 10 blocks of 24 trials each, with all the conditions balanced across blocks and the order of conditions randomized within a block. Participants practiced 8 trials before starting the experiment. The entire experiment was completed in two sessions of 1 hour each, carried out on two different days.

### Data analysis

Two main indexes of interest were computed individually for each of the experimental conditions: Sensitivity to the deviant (*d’*) and the response criterion (*C*), following Signal Detection Theory [33]. *d’* was calculated as the *z*-score transformed hit rate minus the *z*-score transformed false alarm rate, and it measured perceptual sensitivity with which one can discriminate a target (here, a deviant) from the absence thereof. A greater *d’* value indicated greater sensitivity. *C* was calculated as the average of the *z*-transformed hit rate and the *z*-transformed false alarm rate, multiplied by minus one. It indicated whether the decision criterion was biased in one direction or the other. A more positive *C* value reflected more conservative response, i.e., tending more to respond “target absent”. For each index, repeated-measures ANOVA was conducted, in which Greenhouse–Geisser correction was applied to the *p* values of effects of auditory interference. Tukey HSD was used for post hoc tests following a significant main effect. For each significant effect identified by the ANOVA, the estimated Bayes factor [34] was also supplemented to indicate the strength of the effect.

## Results

### Deviant detection

**Fig 3:**
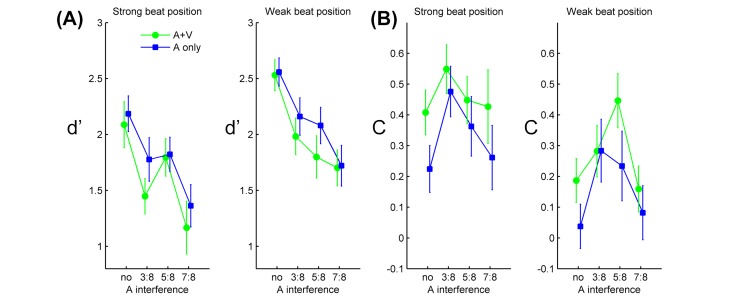
Results of sensitivity (*d’*) and response criterion (*C*). (A) Group means of *d’*, (B) group means of *C*, as a function of the auditory interference level, for each experimental condition separately. Error bars represent standard error of the means.

The individual *d’*s were submitted to a 2 (rhythm modality) × 4 (auditory interference) × 2 (metrical position of deviant) ANOVA. There was a main effect of metrical position, *F*(1, 19) = 50.9, *p* < 0.001, *η*_*p*_^*2*^ = 0.73, showing greater sensitivity to a deviant in the weak beat than in the strong beat positions (Bayes factor 6.5×10^6^: 1 in favor of the effect). The main effect of auditory interference was also significant, *F*(3, 57) = 22.17, *p* < 0.001, *η*_*p*_^*2*^ = 0.54 (Bayes factor 1.7×10^17^: 1 in favor of the effect). Post-hoc comparisons showed greater *d’* without auditory interference compared to all the other conditions with, all three *p*s < 0.001, and also greater *d’* in the 3:8 and 5:8 compared to the 7:8 condition, both *p*s < 0.03. The effect of rhythm modality was marginally significant, *F*(1, 19) = 3.93, *p* = 0.06, *η*_*p*_^*2*^ = 0.17 (Bayes factor 2.3: 1 in favor of the effect), with a trend of somewhat greater *d’* for A than for AV. There was no significant interaction amongst the factors, all *p*s > 0.1. See **Fig 3A**.

As for the response criterion (*C*), the 2 × 4 × 2 ANOVA revealed a significant main effect of rhythm modality, *F*(1, 19) = 6.15, *p* = 0.02, *η*_*p*_^*2*^ = 0.24 (Bayes factor 72: 1 in favor of the effect), showing more conservative response in AV than in A conditions (**Fig 3B**). Although there was also a significant main effect of metrical position, *F*(1, 19) = 50.9, *p* < 0.001, *η*_*p*_^*2*^ = 0.73 (Bayes factor 2.4×10^5^: 1), this effect apparently reflected the results of hit rate and *d’*, as there was no separate false alarm rates for the two metrical position conditions. In short, the effect of metrical position on *d’* supported the PC hypothesis and the idea of enhanced illusory phenomenal accents in strong beat positions. The effect of rhythm modality on *C* revealed more biased response in AV than in A that could also be associated with illusory phenomenal accents: namely, expectations of event saliency were greater in AV due to a stronger metrical accentuation, which shifted the decision criterion to be more conservative.

**Fig 4:**
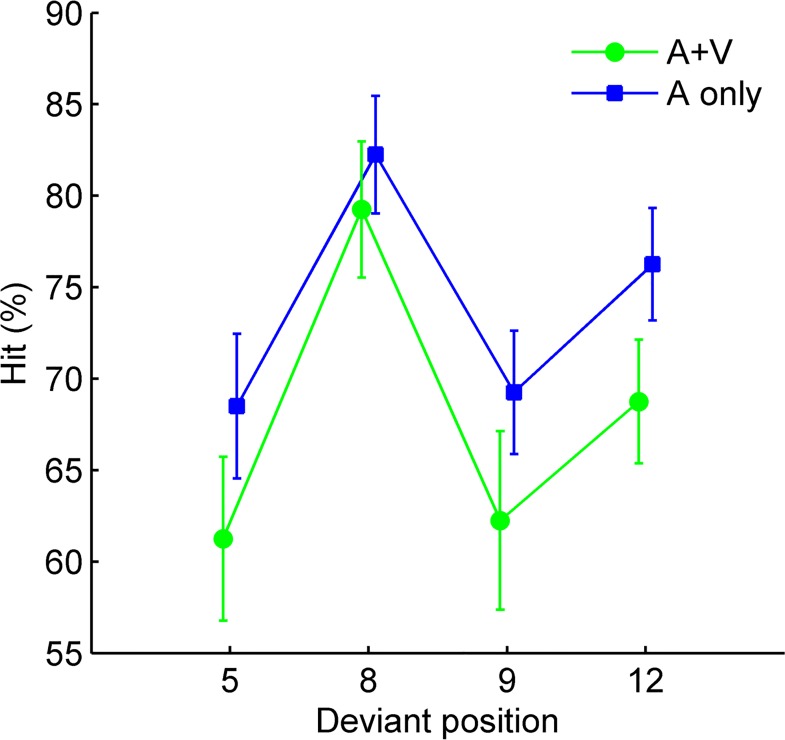
Results of hit rate in each deviant position. Group means of individual hit rates for A and AV conditions are plotted as a function of deviant position. The strong beat deviants occurred in the 5^th^ and 9^th^ pulse positions, whereas the weak beat deviants occurred in the 8^th^ and 12^th^ positions. Error bars represent standard error of the means.

Given the effect of metrical position on *d’*, it is critical to verify whether better detection in weak beat compared to strong beat positions was merely associated with the absolute timing of the deviant occurrence, i.e., whether deviants occurring later in the rhythm were simply better detected. For this purpose, hit rates for each of the four deviant positions (5^th^, 8^th^, 9^th^, and 12^th^ pulse positions), for the A and AV conditions separately, were calculated for every participant. The individual hit rates were then submitted to a 2 (rhythm modality) × 4 (deviant position) ANOVA, which revealed a significant main effect of rhythm modality, *F*(1, 19) = 12.24, *p* = 0.002, *η*_*p*_^*2*^ = 0.39 (Bayes factor 191: 1), with higher hit rates in A than in AV conditions. There was also a significant main effect of deviant position, *F*(3, 57) = 17.74, *p* < 0.001, *η*_*p*_^*2*^ = 0.48 (Bayes factor 1.3×10^9^: 1). Post-hoc comparisons revealed only higher hit rates in position 8^th^ than in position 5^th^ and position 9^th^ (both *p*s < 0.001). There was no interaction between the two factors, *F*(3, 57) = 0.73, *p* = 0.539, *η*_*p*_^*2*^ = 0.04 (**Fig 4**). The pattern showed that deviants occurring in both strong beat positions were consistently less well detected than in the earlier weak beat position (the 8^th^ pulse position). Thus, the hit rate results confirmed that better detection in weak beat positions was *not* due to the advantage of late compared to early deviants, which would have led to a gradual improvement across the four positions.

### Multisensory profile

To examine whether the present multisensory perceptual profile followed the principle of inverse effectiveness, data in the multisensory (AV) compared to unisensory (A) conditions should be described as a function of the response (i.e., task performance) level rather than the stimulus level [35]. This is because, as also reported in the previous study [22], individual *d’*s in the A conditions did not always decline progressively according to the increasing auditory interference, meaning that the auditory perception was not incrementally weakened in the interference order of none to 7:8. Therefore, to characterize the multisensory profile in terms of the performance level, the same analysis procedure as described in [22] was carried out, which involved first sorting each participant’s *d’* in the A conditions in a descending order. This yielded four A performance levels for each participant, representing best to worst auditory detection. For example, instead of “none, 3:8, 5:8, and 7:8”, for a given participant the order could be “none, 5:8, 3:8, and 7:8”. The AV performance level for each participant was then sorted according to the order of the A performance level.

**Fig 5:**
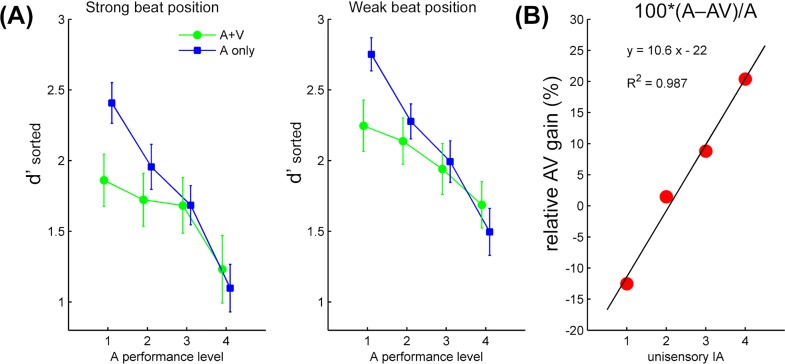
Results of sorted *d’*. (A) Group means of individually sorted *d’*, for each experimental condition as a function of the auditory performance level. 1 to 4 on the *X*-axis represent unisensory (auditory) performance in a descending order. (B) The relative multisensory gain of IA as a function of the unisensory IA level. 1 to 4 on the *X*-axis represent unisensory IA in a descending order. Note that the “gain” here refers to the increase in IA, not in detection performance. The black line depicts the best linear fit for the four mean data points (in red).

The individually sorted *d’*s were then submitted to a 2 (modality) × 4 (A performance level) × 2 (metrical position) ANOVA. While the main effect of metrical position was the same as for the unsorted *d’*: *F*(1, 19) = 50.9, *p* < 0.001, *η*_*p*_^*2*^ = 0.73 (Bayes factor 1.5 × 10^8^: 1), there was an interaction between rhythm modality and A performance level, *F*(3, 57) = 9.86, *p* < 0.001, *η*_*p*_^*2*^ = 0.34 (Bayes factor 331: 1 in favor of the interaction). Follow-up one-way ANOVAs conducted for each performance level separately revealed lower *d’* in AV than in A for level 1 (best A performance), *F*(1, 19) = 19.43, *p* < 0.001, *η*_*p*_^*2*^ = 0.51, marginally so for level 2, *F*(1, 19) = 4.10, *p* = 0.057, *η*_*p*_^*2*^ = 0.18, but not for level 3 or level 4, both *p*s > 0.1. See **Fig 5A**.

The analyses above showed that *d’* was attenuated in the AV compared to A conditions, but more so when *d’* in A was greater. This suggested that the visual rhythm enhanced auditory metrical accents more, leading to more illusory phenomenal accents and thus lower sensitivity to a loudness increment, when there was less such illusory accents (IA) in the auditory rhythm. While this result represented the absolute gain of multisensory accents, it would also be informative to characterize the integration in terms of the *relative* gain [36], i.e., whether the strength of multisensory accents–indicated by the reduction of *d’* in AV from each A baseline–increased *monotonically* across decreasing levels of auditory IA. For this purpose, the percentage of visually enhanced IA was computed on an individual basis, using the *d’* scores averaged across beat positions, as 100 × (A–AV) / A [22,36]. To facilitate visualization, the four mean data points were plotted in a reversed order, such that level 1 to level 4 on the X-axis represented unisensory IA in a *descending* order (i.e., highest to lowest auditory IA, and thus lowest to highest performance in A). The best-fitted linear regression of the group means yielded a strong goodness of fit (*R*^*2*^) of 0.987 (**Fig 5B**), showing a linear increase in multisensory IA across decreasing levels of unisensory IA.

### Magnitude of illusory accent

**Fig 6:**
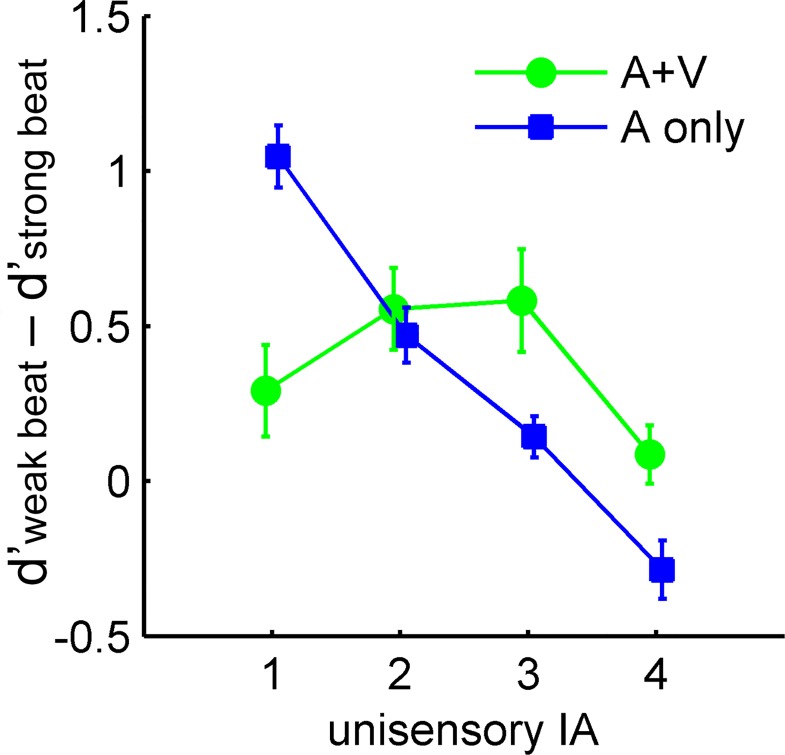
Results of the magnitude of IA. Group means of individually sorted unisensory and multisensory IA as a function of unisensory IA magnitude. 1 to 4 on the *X*-axis represent unisensory IA magnitude in a descending order. Error bars represent standard error of the means.

Following the *d’* results supporting the PC hypothesis and the metrically induced IA, an additional index of the magnitude of IA was calculated for each individual as the difference in *d’* between weak beat and strong beat positions (*d’*_weak beat_−*d’*_strong beat_), for each of the 2 (rhythm modality) × 4 (auditory interference) conditions. A greater value suggested a greater extent of IA that was specifically related to the metrical position. To examine the magnitude of multisensory compared to unisensory IA as a function of the unisensory IA magnitude, the same sorting logic as previously described was applied. Here, each individual’s IA scores in the AV conditions were sorted by the descending order of IA scores in the A conditions. The individual values of sorted IA were submitted to a 2 (rhythm modality) × 4 (auditory IA level) ANOVA, which revealed a significant interaction between the two factors, *F*(3, 57) = 16.35, *p* < 0.001, *η*_*p*_^*2*^ = 0.42 (Bayes factor 8.5× 10^4^: 1 in favor of the interaction). Follow-up partial ANOVAs conducted for each auditory IA level separately showed the following: For level 1 (greatest auditory IA), IA was lower in AV than in A, *F*(1, 19) = 27.28, *p* < 0.001, *η*_*p*_^*2*^ = 0.59. For level 2, IA did not differ between AV and A, *F*(1, 19) = 0.40, *p* = 0.53, *η*_*p*_^*2*^ = 0.02. For level 3 and level 4 (the two lowest auditory IA), IA was greater in AV than in A, *F*(1, 19) = 5.63, *p* = 0.03, *η*_*p*_^*2*^ = 0.23, and *F*(1, 19) = 7.53, *p* = 0.01, *η*_*p*_^*2*^ = 0.28 (**Fig 6**)

In summary, sensitivity to a loudness deviant was greater in weak beat than in strong beat positions, consistent with the PC hypothesis and the idea of metrically induced illusory phenomenal accents. There was more conservative response as well as a trend of reduced sensitivity in the AV compared to A conditions, suggesting greater illusory phenomenal accents in the former. Furthermore, AV stimuli increased IA more (more reduction of *d’* in AV compared to A) when there was less IA in the A conditions (higher *d’* in A). The relative gain of multisensory IA also increased linearly across decreasing levels of unisensory IA, consistent with the principle of inverse effectiveness. Finally, in line with these results, the magnitude of multisensory (relative to unisensory) IA, derived individually from the *d’* difference between metrical positions, increased as the corresponding magnitude of unisensory IA decreased.

## Discussion

This study investigated whether perception of non-isochronous auditory rhythms, probed by sensitivity to a loudness deviant, was consistent with the DAT [13] or the PC account [18], and whether multisensory enhancement of metrical perception would be observed in either case. Participants detected a loudness increment in strongly metrical auditory rhythms while either observing a point-light figure dancing to the rhythm (AV), or without any visual stimuli (A), both in the presence or absence of an auditory interference. Results showed that sensitivity to a deviant was greater in weak beat than in strong beat positions across all conditions, supporting the PC hypothesis and the effect of metrically induced illusory phenomenal accents. The presence of the visual rhythm–communicated by the dance movement–increased auditory IA and consequently attenuated sensitivity to the auditory deviant, but more so when the auditory sensitivity was itself less affected (i.e., less auditory IA). In addition, multisensory enhancement of IA (AV compared to A) was greater when the unisensory IA was weaker, which was shown in both the absolute and the relative gain, as well as in the individually indexed magnitude of IA. This pattern suggested that multisensory metrical information between the auditory and the visual rhythms can be integrated, following the principle of inverse effectiveness, to reinforce perception of illusory phenomenal accents.

The finding that sensitivity to a loudness increment was greater in weak beat than in strong beat positions is consistent with that reported by [12] (in the first experiment), but contradicted by the results of [7]. Both previous studies adopted isochronous auditory stimuli, where metrical accents were induced by subjective accentuation. In the former, participants listened to an isochronous sequence without further instruction, and the percept of a duple meter was supposed to emerge naturally [37]; in the latter, participants were required to mentally impose a triple meter while listening. As the present study presented rhythms of a duple meter and the result conformed to that of the former, it might suggest that the hierarchical temporal structure of a duple meter is more readily represented in the listener than a triple meter, which facilitates predictive computation. This is corroborated by findings of a perceptual advantage in rhythmic timing for duple compared to triple meters [38]. While subjective accentuation of an isochronous sequence in both meters can entrain neural responses accordingly [39], there is evidence that motor areas of the brain implicated in beat perception, such as basal ganglia [40], react more strongly to the downbeat in a duple than in a triple meter [41]. In light of the PC account, the motor system is proposed to send top-down signals to facilitate auditory temporal prediction [42]. Thus, rhythms that yield a clearer duple meter may couple the motor system more effectively, allowing the perceptual system to make stronger predictions based on the metrical structure. In a similar vein, metrically induced illusory phenomenal accents may be attributed to the same top-down modulation [43]: It has been shown that the percept in strong beat positions is more reinforced by motor signals compared to that in weak beat positions. More importantly, the motor-related neural response (e.g., beta-band oscillations) under subjective accentuation resembles that elicited by a real loudness increase [43,44], which could indeed serve the neural substrate of illusory phenomenal accents. As such, an increase in loudness in strong beat positions needs to exceed a higher threshold in order to be perceived as louder.

A few points are worth addressing regarding the present effect of metrical position on deviant detection. Because non-isochronous rhythms were presented in this study, one question arises as to whether the effect might have been associated with different temporal or acoustic contexts preceding strong beat and weak beat deviants, e.g., whether either of them was more likely preceded by an event or by silence. As seen in **Table 2**, in five of the ten rhythms the two deviant positions had a similar preceding context (both preceded by silence or both by an event). In the rest of the rhythms the frequency of either deviant position being preceded by silence was largely balanced (two for the weak beat deviant and three for the strong beat one). It thus seems unlikely that the temporal contexts preceding the two deviant types were different enough to account for the detection result. Another similar concern is whether the two deviant positions had different temporal relations to the interference sequences that could affect detection. The obtained data, however, showed no interaction between the metrical position and the auditory interference level (**Fig 3**), and the latter included a condition without any interference. Therefore it seems reasonable to assume that, while the temporal distances between interference tones and the two deviant positions varied across different rhythm-interference combinations, it did not appear to modulate the result.

The other main result, i.e., multisensory metrical perception when watching a humanlike figure dance to the heard rhythm, extends the previous finding involving a simple periodic movement [22] into a more complex and ecological scenario. Here, the dance movement encompassed a metrical structure that clearly matched the meter of the auditory rhythm. As visual rhythm perception of dance has been proposed to engage similar mechanisms to auditory rhythm perception of music [25,31,45], the present results further suggest that the rhythms of both can be perceptually integrated. AV integration in this case strengthened the perceived metrical structure, giving rise to more illusory phenomenal accents [20] as compared to the auditory baselines. Moreover, metrical integration appeared to follow the inverse effectiveness principle, such that multisensory IA increased linearly across decreasing levels of unisensory IA. A similar pattern was also observed in the IA magnitude as indexed individually by the sensitivity difference between weak beat and strong beat positions. In other words, the less IA-inducing the auditory rhythm, the more the visual rhythm can increase IA by enhancing metrical accentuation. Interestingly, the relative multisensory gain observed here abided the principle of inverse effectiveness more closely than that in the previous study, where a similar paradigm was employed to probe sensitivity to a temporal deviant in auditory rhythms [22]. This may be attributed to several factors: For one, the present visual stimuli were more naturalistic and fitting to the musical rhythm [25], and the match in content likely promoted AV integration [46]. Besides, the multisensory integration profile is originally derived from the firing rate of cats’ superior colliculus neurons [27], whereby both the uni- and multisensory response magnitudes are proportional to the stimulus intensity. When measuring human response to complex AV information, such as speech or the present musical stimuli, the response level does not necessarily scale with the stimulus strength [22,29], and both the task and the analysis method are found to affect whether the data obey the integration principles [29,36]. That the present results followed the inverse effectiveness principle argues that not only can the auditory and visual rhythms of music and dance be integrated, but also that the inverse effectiveness can be used to describe illusory percepts as a result of this integration. While different measures of illusory percepts have long been employed to indicate cross-modal binding in speech (e.g., McGurk effect, see [47] for an overview), the present study revealed the effect parametrically in a musical context. In contrast to AV speech (sounds paired with lip movement), integration between music and dance rhythms can occur without the sounds being consequent upon the viewed action.

While the observed multisensory profile indicated integration of the AV rhythms, the lack of an interaction between metrical position and rhythm modality in the sorted *d’* (**Fig 5**) might suggest that the visual rhythm added accents (or expectations) not only in the strong beat positions. As two concurrent levels of periodicity existed in the visual rhythm, i.e., the more salient periodicity of the leg movement and the less salient one of the bounce, it could be that both levels were integrated with the auditory rhythm. That is, the degree of (illusory) accents or expectations increased overall in both the auditory strong beat and weak beat positions. Given that no previous study has looked into integration of AV rhythms as complex as the present ones, especially with the visual rhythm derived from biological motion, it remains to be seen in future research whether other forms of naturalistic visual rhythms may accentuate only a specific level of the auditory rhythm. Nevertheless, the present study revealed that the individually indexed sensitivity difference between strong and weak beat positions (i.e., the extent of illusory phenomenal accents) in AV conditions increased as the corresponding difference in A conditions decreased (**Fig 6**), which indicated integration as being modulated by metrical position. Across individuals, this index might not have followed the same order as the stimulus interference level or the auditory performance level. One way to better understand the relationship amongst these factors in the future would be to present stimuli that yield the same order of performance level as the imposed level of stimulus strength, though this might prove challenging as far as complex stimuli are concerned [29].

How does the visual movement rhythm enhance auditory metrical perception? One possible mechanism is that AV integration is also governed by the PC principle [48]. As often proposed in the speech domain, visual information derived from a speaker’s facial or lip motion can serve as a *prior* to predict the auditory events [24,49]. Here, similarly, the dance movement corresponded to the metrical structure of the auditory rhythm, and the visual cue may thus be adopted to enhance prediction of the latter [46]. Furthermore, when the information consists of a regular temporal structure, such as the present AV rhythms, the predictive mechanism operates for both “what” (i.e., is the event accented?) and “when” (i.e., when is an event accented?), both of which could implicate the motor system. In terms of predicting “what”, the motor system may reinforce the perceived accents of an auditory rhythm by simulating periodic motion internally, which then feeds back to modulate auditory perception [43,50]. Such motor simulation may be evoked not only by hearing the musical rhythm, but also by visually observing a dance movement that matches this rhythm [25,51]. In terms of predicting “when”, the motor system also appears instrumental in predictive timing of rhythmic streams [52]: Rhythmic motor activities are proposed to reset oscillations of the sensory cortices, such that the phase of neural excitability would temporally align with the external rhythmic events to enhance stimulus processing [42]. As action observation and action execution share overlapping neural substrates in the motor system [53], covert motor activities through observing rhythmic dance movements might modulate auditory processes in a similar manner. Finally, a recent study shows that both the imagined and the physically imposed auditory metrical accents elicit greater event-related desynchronization in the cortical beta oscillations [44]. This decrease in beta power is considered a neural marker of motor activities following both the executed as well as the observed movements, including rhythmic limb motions [54]. As such, dance observation in the present task may have led to similar beta activities, which modulated auditory processes and enhanced the illusory phenomenal accents in a similar manner as the imagined accents.

In conclusion, the present study provides support for the PC mechanism as underlying perception of strongly metrical auditory rhythms, a result also consistent with metrically induced illusory phenomenal accents. Besides, the perceived metrical structure–and hence the IA–in these rhythms can be enhanced by simultaneously observing a dance movement with a matching meter. The obtained pattern of AV metrical perception follows the principle of inverse effectiveness, suggesting integration of rhythm information between (auditory) music and (visual) dance. As the integration of AV rhythms involving movement observation can also be accommodated in the PC framework, the visual input may modulate auditory processes through motor mediation. Several questions arising from these results are worth addressing in the future, such as the effect of metrical congruency between music and dance on AV integration, the extent of AV integration in a duple compared to a triple meter, or indeed the neural correlates (e.g., beta oscillations) of these effects.

## Supporting Information

S1 Audio Auditory rhythm (2112231). This is the same rhythm as illustrated in Fig 2.
(WAV)

S2 Audio Auditory rhythm (2112231) in the presence of an auditory interference. The beat ratio between the two is 3:8 (8 being that of the rhythm).
(WAV)

S3 Audio Auditory rhythm (2112231) in the presence of an auditory interference. The beat ratio between the two is 5:8 (8 being that of the rhythm).
(WAV)

S4 Audio Auditory rhythm (2112231) in the presence of an auditory interference. The beat ratio between the two is 7:8 (8 being that of the rhythm).
(WAV)

S1 Table Dataset of the individual *d’* and *C* values. Data are arranged by experimental condition (in columns) and participant number (in rows).
(XLSX)

S1 Video Visual stimuli. Four looped cycles of the point-light dance movement are shown in this video.
(MP4)
